# Curcumae Radix Extract Decreases Mammary Tumor-Derived Lung Metastasis via Suppression of C-C Chemokine Receptor Type 7 Expression

**DOI:** 10.3390/nu11020410

**Published:** 2019-02-15

**Authors:** Pelin Kaya, Sang R. Lee, Young Ho Lee, Sun Woo Kwon, Hyun Yang, Hye Won Lee, Eui-Ju Hong

**Affiliations:** 1 College of Veterinary Medicine, Chungnam National University, Daejeon 34134, Korea; pelinkaya.217@gmail.com (P.K.); srlee5@naver.com (S.R.L.); lee05@cnu.ac.kr (Y.H.L.); ksunwoo12@gmail.com (S.W.K.); 2 Herbal Medicine Research Division, Korea Institute of Oriental Medicine, Daejeon 34054, Korea; hyunyang@kiom.re.kr

**Keywords:** breast cancer, curcumae radix, PyMT/MMTV, CCR7, MMP9

## Abstract

Curcumae radix is the dry root of *Curcuma longa L.* (turmeric) that can be used either as a spice or traditional medicine. The aim of this study was to investigate the survival benefits and the anti-metastatic activity of curcumae radix extract (CRE) in MCF7 cells and in MMTV-PyMT transgenic mice—a mouse model of breast cancer metastasis. In vitro wound scratch assay revealed that CRE treatment inhibited cell motility and cell migration in a dose-dependent manner. To investigate the effect of CRE in breast cancer metastasis, MMTV-PyMT transgenic female virgin mice were used and randomly divided into two groups. For survival curve analysis, CRE was administered in a dose of 50 mg/kg to 8–20-week-old mice. Interestingly, CRE treatment significantly increased the median and prolonged survival of MMTV-PyMT mice. Furthermore, CRE treatment decreased tumor burden and inhibited cell proliferation in primary breast tumor, and also suppressed mammary tumor-derived lung metastasis. The size of the lung metastases substantially decreased in the CRE-treated group compared with the ones in the control group. Curcumae radix extract showed anti-metastatic activity through regulating the expression of metastasis markers including C-C Chemokine Receptor Type 7, Matrix Metalloproteinase 9 and the proto-oncogenes c-fos and c-jun. We demonstrated that these metastatic regulators were decreased when CCR7 expression was suppressed in MCF7 cells transfected with CCR7 siRNA. The results of this study show that curcumae radix exerts antitumor and anti-metastatic activities, and we suggest that curcumae radix might be a potential supplement for the treatment and prevention of breast cancer metastasis.

## 1. Introduction

Breast cancer is the leading cause of cancer-related death among women worldwide and is the most frequently diagnosed cancer; in 2018, one in four cancer cases were diagnosed as breast cancer among women. Over 90% of mortality cases are due to distinct metastatic patterns of breast cancer including lung, bone marrow, regional lymph nodes, and liver [1,2]. Metastatic breast cancer (stage IV breast cancer) represents a major clinical problem as it is difficult to be surgically removed, unlike the primary tumor. In particular, the lungs and bones are among the most frequent target sites of metastatic breast cancer. Despite new developing technology and medical conditions, there is currently no effective cure for metastasis [3,4].

Curcumae radix is the dry root of *Curcuma longa L.,* which is a plant belonging to the ginger family that is used as traditional medicine in South and Southeast Asia [5,6]. Recently, it has caught attention for having diverse pharmacological effects including anti-inflammatory [7], antiviral, antimicrobial [8,9], and anticancer effects [10,11,12,13], as demonstrated by several reports. Curcumae radix and its active compounds, such as curcuminoids, exert an anticancer role by regulating multiple intracellular signaling pathways involving proliferation, apoptosis [14,15], cell phase-related genes, immune system, microRNAs [16], and telomerase activity [17]. It has been reported that demethoxycurcumin, one of the active component in *Curcuma longa L.*, suppresses cell migration and invasion by targeting Nuclear Factor-κB (NF-κB) in MDA-MB-231, a human breast cancer cell line [18]. Another active component in *Curcuma longa L*., curcumin, exhibits potent anticancer and anti-metastatic effects [19,20] by modulating the NF-κB signaling pathway and matrix metalloproteinase (MMPs) inhibitors in the MCF7 breast cancer cell line [21] and in a nude mice animal model [22]. Another study indicated curcumin as a favorable anticancer agent for enhancing the efficacy of chemotherapy [23]. Moreover, it has been reported that diterpenoid C of curcumae radix inhibited proliferation in human colon adenocarcinoma cells by inhibiting the mitogen-activated protein kinase (MAPK) signaling pathway [24].

Until now, there have only been a few in vivo studies with curcumae radix and all of the studies reported in the literature were focused on gastric cancer [25,26]. Although there are several studies about the effect of curcumin on breast cancer, the antitumor and anti-metastatic effect of Curcumae radix extract on breast cancer is still unknown. In this study, we have employed MMTV-PyMT (mouse mammary tumor virus-polyoma virus middle t antigen) transgenic mice to directly investigate the effects of ethanol extract of curcumae radix in tumor development and lung metastasis of breast cancer. Mouse mammary tumor virus -PyMT transgenic mouse model mimics human breast cancer, developing palpable mammary tumors and metastasizing to lung and lymph. Our in vivo model provides the advantage of high penetrance of the early onset of mammary cancer, with the primary tumor formation starting around six weeks of age followed by tumor development and lung metastasis, which can be studied without discontinuity [27]. The development of extensive lung metastasis makes MMTV-PyMT mice an ideal model to investigate the role of specific genes in breast cancer. The role of Steroid receptor coactivator-1 (*Src-1*) and Caveolin-1 (*Cav-1*) genes in tumor progression and lung metastasis of breast cancer was observed in MMTV-PyMT mice model. It has been reported that the disruption of *Src-1* gene suppresses tumor progression [28], while the disruption of *Cav-1* showed reverse effects [29]. 

In the present study, the antitumor and anti-metastatic effects of Curcumae radix extract were evaluated. During a survival analysis and 13-week observation period, PyMT transgenic female mice were examined for tumor onset, malignancy, and metastasis. We demonstrated that Curcumae radix extract significantly prolongs the overall survival of PyMT-MMTV mice. As such, this is the first demonstration that Curcumae radix extract administration shows anti-metastatic effects on breast cancer in an in vivo animal model.

## 2. Materials and Methods 

### 2.1. Preparation of Curcumae Radix Extract

Curcumae radix was purchased from Beneherb Agricultural Co. Ltd., Jeju Island, Republic of Korea. The botanical origin plants were deposited in the Herbal Medicine Research Division of Korea Institute of Oriental Medicine (KIOM) in Daejeon, Republic of Korea (voucher specimen KIOM M 130110). Dried Curcumae radix was extracted with 70% (v/v) ethanol by sonication for 120 min. The extracted 70% ethanol solution was filtered through filter paper (Whatman No. 2), and then concentrated using a vacuum rotary evaporator (Büchi; Flawil, Switzerland) at 40 °C. The extracted sample was lyophilized using a freeze-dryer (IlShin; South Korea). The final powder of the 70% ethanol extract of Curcumae radix was 249.2 g (yield, 12.49%). Fifty milligrams Curcumae radix extract (CRE) was dissolved in 1 ml dimethyl sulfoxide (DMSO) solvent (Sigma–Aldrich, Co., St. Louis, MO, USA) and used as a stock solution for in vitro studies.

### 2.2. Quantitative Analysis of Marker Compounds in Curcumae Radix Extract

**Figure 1:**
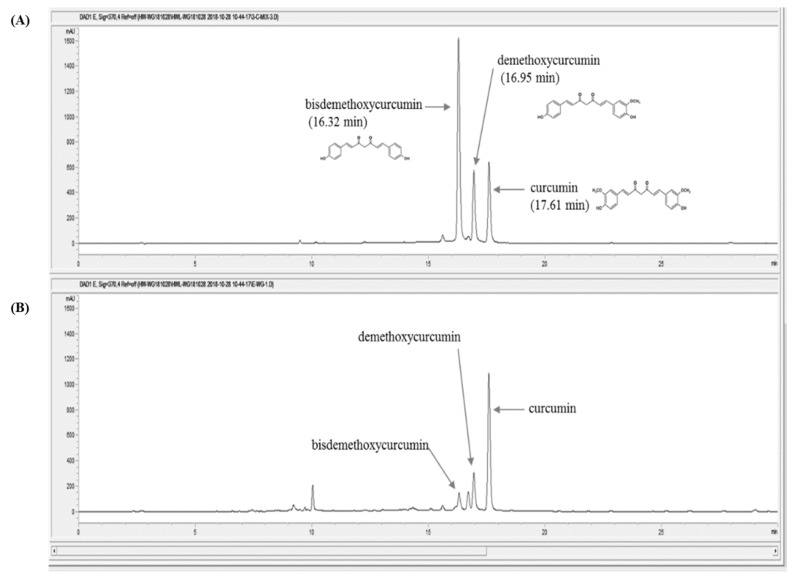
High-performance liquid chromatography (HPLC) chromatograms of Curcumae radix extract and three reference compounds at 370 nm. (**A**) Three reference standards (curcumin, demethoxycurcumin, and bisdemethoxycurcumin), (**B**) Curcumae radix ethanol extraction.

For the quantitative analysis of the marker compound in Curcumae radix, the 70% ethanol extract (50.1 mg) was dissolved in 2 mL of 70% methanol and filtered through a 0.2-μm syringe filter. The Curcumae radix extract sample and three reference compounds: curcumin, demethoxycurcumin, bisdemethoxycurcumin, were analyzed three times by reverse-phase using a 1100 series high-performance liquid chromatography (HPLC, Agilent Technologies, Santa Clara, CA, USA). The analytical column with a Kinetex C18 (4.6 × 250 nm, 5 μm, Phenomenex) was used as the gradient phase and was maintained at 30 °C during the experiment. The mobile phase was composed of distilled water in 0.1% formic acid (Figure 1A) and acetonitrile (Figure 1B). The gradient flow was as follows: 0–5 min, 20%–40% (*v*/*v*) B; 5–15 min, 40%–50% (*v*/*v*) B; 15–20 min, 50%–60% (*v*/*v*) B; 20–30 min, 60%–70% (*v*/*v*) B; 30–50 min, and 70–100% (*v*/*v*) B. The PDA detector wavelength of the reference compounds were recorded at 370 nm with injection volume of 10 μL and a flow rate of 1.0 mL/min. The acquisition and process of retention time and UV spectra data was carried out with ChemStation software (Agilent Technologies, Santa Clara, CA, USA).

### 2.3. Cell Culture and Transfection

All cells were cultured in Dulbecco’s modified Eagle’s medium (DMEM) supplemented with 5% fetal bovine serum, 100 units/mL penicillin, and 100 µg/mL streptomycin. Cell culture plates were maintained in humidified incubators at 37 °C in a 5 % CO_2_ incubator. Negative control siRNA and CCR7 siRNA were purchased from GenePharma (Shanghai GenePharma Co., Ltd. Shanghai, China). The sense sequence of CCR7 siRNA was 5′-GCG UCA ACC CUU UCU UGU ATT-3′ and control siRNA was 5′-UUC UCC GAA CGU GUC ACG UTT-3′. Small interfering RNA (siRNA) transfection was performed by using lipofectamine 2000 (11668-027, Thermofisher) according to the manufacturer’s protocol. 

### 2.4. Animal Studies

In this study we used hemizygous FVB/NTgN (MMTVPyVT) 634 Mul (Muuler WJ) female virgins and non-transgenic control mice. Transgenic mice were purchased from the Jackson Laboratory (002374). All animals were housed in a pathogen-free facility at Chungnam National University under a 12 hours standard light/dark cycle and fed with ad libitum access to standard chow and water. All mouse experiments were performed according to Chungnam National University Facility Animal Care Committee standards. Hemizygous female MMTV-PyMT mice were generated by breeding Hemizygous male and non-transgenic female mouse. All animals were tagged and tail-clipped at age of 4 weeks, genotyped by PCR using a primer pair specific for the MMTV-PymT transgene, and distributed randomly into the treatment groups. 

### 2.5. Animal Treatment and Excision of Organs

Hemizygous MMTV-PyMT female virgins were randomly assigned to different treatment groups (6–7 were included in each group). Animals were palpated twice weekly beginning at six weeks of age to check the development of tumors. At a dose of 50 mg/kg Curcumae radix extract was administered orally 5 times in a week. Tap water was used as a solvent for Curcumae radix extract. Subsequently, control group were treated with 1 ml tap water same as CRE treatment. For survival experiment, CRE administration was started at 8 weeks of age and continued until death of animal. For short term analysis, CRE administration was started at 6 weeks of age. Beginning at 13 weeks of age, female mice were sacrificed and all tumors and lung were carefully excised and weighed. Portions of the tumors and organs were also stored in formalin for fixation purposes. 

### 2.6. Histological Analysis of Tumor and Lung Tissue

Mammary tumors and lung samples were excised, cut into smaller portions, and fixed with 10% buffered formalin for over 72 hours then embedded in paraffin. Sections were cut at 4 microns and attached to silane coated slides, stained with hematoxylin and eosin at Histological laboratory of the Comparative Animal Resource Center at Chungnam National University. Stained slides were examined by using Leica DM 3000 LED microscope and then images were evaluated. The primary antibody anti-Ki 67 (Abcam, Cambridge, UK) and anti-Ccr7 (#NBP2-67324, Novus Biologicals, USA) were used for Immunohistochemisrty staining. Slides were displayed and photographed under microscope (HCImage Live, Hamamatsu Photonics K.K, USA) to evaluate Ki67 and Ccr7 proteins. Image processing was done by using ImageJ software (National Institutes of Health, Bethesda, MD, USA).

### 2.7. RNA Analysis and Reverse Transcription-PCR 

Mouse tumor and lung samples homogenized in TRIzol® reagent, and total RNA was extracted according to the instructions of the manufacturer (Ambion, Life technologies, Carlsbad, CA, USA). Mcf7 cells 2 × 10^6^ were seeded to 6-well plates. Curcumae radix treatment was done when the cells reach 60–70% confluency. Dimethyl Sulfoxide (DMSO) solvent (Sigma–Aldrich, Co., St. Louis, USA) was used as vehicle control. Medium was refreshed every 12 hour. At the end of 48 hours, cells were suspended in TRIzol reagent and same protocol was applied. Then, cDNA was synthesized with 1.5 μg of total RNA and Excel RT Reverse transcriptase kit (RP1300, SMOBIO, Hsinchu City, Taiwan). Amplification of cDNA was done by RT-PCR using AmpliTaq Gold DNA polymerase and quantitative real-time PCR. Amplification of cDNA was done by using Sybr Green Q-PCR mix (Smartgene, SamJung Bioscience, Daejeon, Korea) by Stratagene Mx3000P QPCR systems (Agilent Biotechnologies, Santa Clara, United States). Primers were synthesized by Macrogen Inc. (Seoul, Korea) and Bionics CRO (Seoul, Korea) (Table 1). Gene expressions of β-ACTIN and GAPDH were used as the control gene for Mcf7 cells. Hprt gene expression was used as a control in tumor and for lung samples the *β-actin* gene was used as a housekeeping gene. All experiments were repeated at least three times. Fold change in gene expression was calculated based on the cycle threshold and amplification curves were used to monitor mRNA values.

### 2.8. Protein Analysis and Western Blotting

One-hundred-and-fifty to 200 mg portions of mouse tumor and lung samples homogenized in 700 µL T-PER^TM^ tissue protein extraction reagent (Thermo scientific, USA) that consisted of proprietary detergent in 25 mM bicine and 150 mM sodium chloride, pH 7.6. Protease inhibitor phenylmethylsulfonyl fluoride (PMSF, Sigma–Aldrich, Co., St. Louis, Mo, USA) was added to protein extraction reagent before use. For cell experiments, 2 × 10^6^ Mcf7 cells were seeded to 6-well plates. Curcumae radix extract treatment with different concentrations was done when the cells reach 90% confluency and DMSO was used as vehicle control in experiments. Medium was refreshed every 24 hour. At the end of 48 hours, cells were suspended in 400 µL T-PER^TM^ Tissue Protein extraction reagent (Thermo scientific, USA) that contains protease inhibitor phenylmethylsulfonyl fluoride (PMSF, Sigma–Aldrich, Co., St. Louis, MO, USA). Homogenized samples were centrifuged at 13,000 rpm for 15 min. Supernatant was taken to pre-cooled new 1.5 ml microcentrifuge tubes. Sonication was applied by using sonicator (QSONICA, USA). Quantification of homogenized samples was done by Bradford assay with PRO-Measure solution (Intron Biotechnology, 21011, Korea) and then homogenized samples incubated at 100 °C for 5 min. Total protein samples were loaded to 8% and 10% SDS-PAGE gels, proteins were transferred to nitrocellulose membranes with Bio-Rad Power Pac in 350 mA. Membranes were blocked with 3% skim milk then incubated with specific primary antibodies for overnight at 4 °C. After washing with TBS-T, membranes were incubated with corresponding horseradish peroxidase (HRP)-conjugated secondary antibody (AE-1475 goat anti-rabbit, BS-0296G-HRP goat anti-mouse, Bioss) for 4 h. Protein bands were detected with ECL solution (XLS025-0000, Cyanagen) and quantification was done with Chemi Doc (Fusion Solo, Vilber Lourmat). The primary antibodies used in the Western blots were as follows: CCR7 antibody (SR36-04) (#NBP2-67324, Novus Biologicals, USA), ERα (2Q418) (#SC-71064, SantaCruz, CA, USA), Phospho-AKT (Ser473) (D9E) XP^®^, pAMPK (Thr172) (40H9), Her2/ERbB2 (D8F12) XP^®^ (Cell Signalling Technology, Inc. Danvers, MA, USA), β-Actin (N21) (#sc-130656, Santacruz, CA, USA), and Gapdh (60004-1-Ig) (ProteinTech Group, Inc.). Rabbit polyclonal antibody for Ccr7 (#sc-130656) was purchased from Santa Cruz biotechnology (Santacruz, CA, USA). Mouse monoclonal antibody specific for G (#sc-13551) was purchased from Santacruz. All the assays using CCR7 knockdown MCF7 cells were performed after 48 h of siRNA transfection.

### 2.9. Cell Viability Assay

Cell viability assay was done by using WelCount™ Cell Viability Assay Kit (#TR005-01, WELGENE). It is a product based on XTT (2,3-Bis (2-methoxy-4-nitro-5-sulfophenyl) -2H-tetrazolium5 carboxanilideinner salt) that measures the number of live cells through a spectrophotometer. Approximately 1 × 10^6^ Mcf7 cells were seeded to. After 24 hour, the XTT assay kit was applied to the control plate to determine cell viability before CRE treatment as 0 hour. At the same time, remaining experiment plates were treated with different concentrations of CRE, and DMSO solvent (Sigma–Aldrich, Co., St. Louis, MO, USA USA) was used as vehicle control. After 24, 48, and 72 h of CRE treatment, cell viability was determined by following the XTT assay kit protocol. After adding XTT reagent, cells were incubated at 37 °C CO^2^ incubator for 2 h. Absorbance was measured at 450 nm using a plate reader. All experiments were performed in triplicate. 

### 2.10. Scratch Wound Healing Assay 

Human breast adenocarcinoma Mcf7 cells were seeded into 24-well plates. When the cell confluence reached about 80%, a line was scratched with a 10 µL plastic pipette tip. The cells were washed three times with DBPS to remove cell debris around line. The cells were scratched and photographed at 0 h then the cells were allowed to migrate in the presence of 25 μg/mL and 40 μg/mL CRE at 37 °C in 5% CO_2_ atmosphere. A DMSO solvent was used as vehicle control. Every eight hours, wounded areas were displayed and photographed under microscope (HCImage Live, Hamamatsu Photonics K.K, Hamamatsu, Japan) to assess cell migration ability. Image processing was done by using ImageJ software (National Institutes of Health, Bethesda, MD, USA). 

### 2.11. Statistical Analysis

All in vitro experiments were reported as means ± SD for at least three independent times. In vivo experiments were shown as means ± SEM. Quantitative results were analyzed by Student’s *t*-test using Graph Pad Software (GraphPad Inc., San Diego, CA, USA).

## 3. Results

### 3.1. Quantitative Analysis of Marker Compounds in Curcumae Radix Extract

Curcumae radix extract and three reference compounds named curcumin, demethoxycurcumin, and bis-demethoxycurcumin, were subjected to HPLC-PDA (high-performance liquid chromatography coupled with a photodiode array UV detector) analysis (Figure 1). The linearity of the HPLC analysis method was estimated by the correlation coefficients values (*r*^2^) based on the calibration curves of the three reference compounds. The three marker compounds curcumin, demethoxycurcumin, and bis-demethoxycurcumin, were finally detected from Curcumae radix at the retention time of 17.60 min, 16.95 min, and 16.32 min, respectively. The concentrations of the three major compounds in Curcumae radix extract are shown in Table 2. 

### 3.2. Curcumae Radix Extract Inhibits Cell Growth, Cell Motility, and Cell Migration of MCF7 Cells 

**Figure 2:**
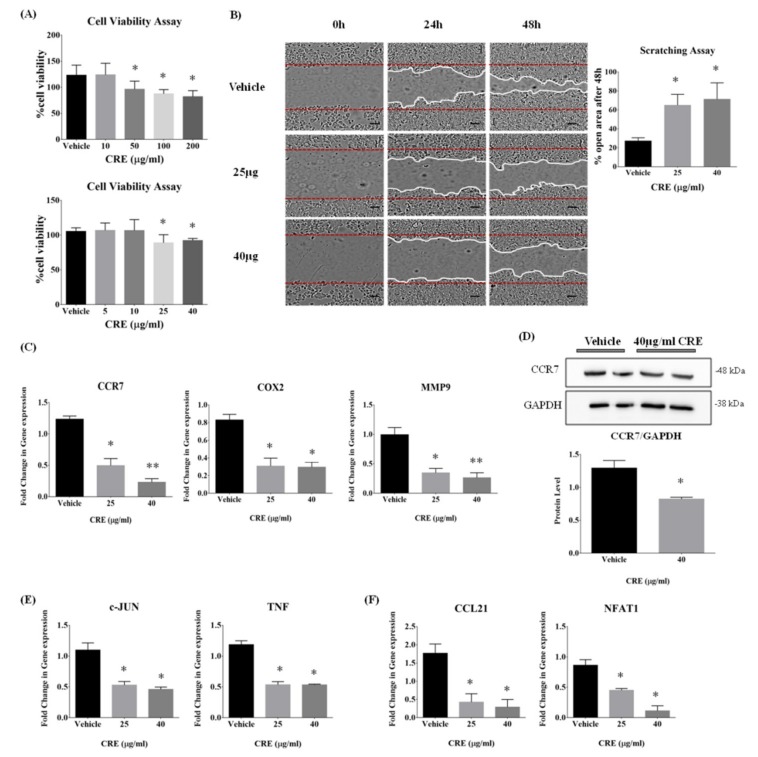
Anti-migration effects of Curcumae radix extract on human breast cancer MCF7 cells. (**A**) Cells were incubated with broad and narrow range of CRE concentrations and cell viabilities were measured by XTT assay at 72 h. Concentrations up to 25 µg/mL Curcumae radix extract did not significantly change cell viability in vitro. Absorbance was expressed as A450 nm. Data are representative of three independent experiments and are expressed as the mean ± SD; * *p* < 0.05 vs. vehicle. (**B**) Scratch wound healing assay was performed with MCF7 cells in the two doses of Curcumae radix extract. Representative images were taken at 0, 24, and 48 h after wound scratch. Red horizontal lines indicate wounded area borders at 0 hour. White lines indicate wounded area borders at 24 and 48 h. Scale bars = 100 px. The graphic represents quantitative analysis of cell migration of MCF7 cells by calculating percentage of unrecovered area after 48 hours by using Image J analysis. We can observe that CRE treated cells slowed down the migration and closed the wound slower than control group. Data are representative of three independent experiments and are expressed as the mean ± SD; * *p* < 0.05 vs. vehicle. (**C**) To investigate level of transcripts associated with breast cancer metastasis, MMP9, CCR7 and COX2 expression levels were monitored in MCF7 cell incubated with CRE. Glyceraldehyde 3-phosphate dehydrogenase (GAPDH) mRNA was used as an internal control in real time PCR. Data are representative of four independent experiments and are expressed as the mean ± SD; * *p* < 0.05 and ** *p* < 0.005 vs. vehicle. (**D**) MCF7 cells were maintained in medium containing 40 µg/mL CRE. Levels of CCR7 protein, a metastasis marker, was analyzed by Western blot. GAPDH was used as an internal control. Values represent means ± SD of at least 3 experiments; * *p* < 0.05 vs. vehicle. (**E**) To investigate the level of transcripts associated with apoptosis and inflammation, c-JUN and TNF-α were monitored in MCF7 cell incubated with CRE. GAPDH mRNA was used as an internal control in real time PCR. Data are representative of three independent experiments and are expressed as the mean ± SD; * *p* < 0.05 vs vehicle. (**F**) To investigate level of transcripts associated with metastasis markers, CCL21 and NFAT1 were monitored in MCF7 cells incubated with CRE. GAPDH mRNA was used as an internal control in real time PCR. Values represent means ± S.E.M. *n* = 3, * *p* < 0.05 vs. vehicle.

Breast cancer MCF7 cells were incubated with Curcumae radix extract and cell viability was evaluated by XTT assay after 72 h. Firstly, broad ranges of CRE concentrations were applied to the cells; then, cell viability was examined in a narrower range. As shown in Figure 2A, the cell viability did not significantly change at concentrations up to 25 µg/mL CRE; however, cytotoxic effects were shown at concentrations ≥25 µg/mL. To evaluate impact of CRE on cell mobility and cell migration of MCF7 cell, scratch wound healing assay was performed using two doses of Curcumae radix extract, 25 µg/mL, and 40 µg/mL. According to the scratch wound healing assay (Figure 2B), the width of the scratched area after 48 hours was significantly larger in CRE treated cells, compared with the control cells. Moreover, Curcumae radix extract inhibited cell motility and cell migration in MCF7 cells in a dose-dependent manner. 

### 3.3. Curcumae Radix Extract Inhibits CCR7, MMP9, and COX2 Gene Expression in MCF7 Cells

Although the exact pathway of breast cancer metastases is unknown, recently several markers correlated with metastasis have been identified through a genome-wide expression analysis by large-scale studies [3,30]. In order to determine which signal paths and genes are affected by CRE and leading to anti-metastatic effects on MCF7 cells, we further investigated several key regulators. There was a significant change at transcriptional levels of metastasis-related genes; specifically, *COX2* (Cyclooxygenase 2), *CCR7* (C-C Chemokine Receptor Type 7), and *MMP9* (Matrix Metalloproteinase 9) mRNA levels (Figure 2C) were significantly decreased 48 hours after the CRE treatment, in a dose-dependent manner. Consistent with the mRNA results, there was also a reduction at CCR7 protein level (Figure 2D). In addition to the metastasis markers, CRE inhibited the gene expression of *c-JUN* (JUN proto-oncogene, AP-1 Transcription Factor Subunit) and TNF (Tumor Necrotic Factor), which play a role in cell apoptosis and cell inflammation, respectively (Figure 2E). As shown in Figure 2F, CRE significantly reduced the mRNA expression level of CCL21 (C-C motif Chemokine Ligand 21), which has a receptor-ligand relationship with CCR7 [31]. The mRNA level of NFAT1 (Nuclear factor of activated T cells 2) was also decreased, inducing COX2 expression and triggering cell invasive migration [32].

### 3.4. Ethanol Extract of Curcumae Radix Significantly Enhances the Survival of MMTV-PyMT Female Transgenic Mice

**Figure 3:**
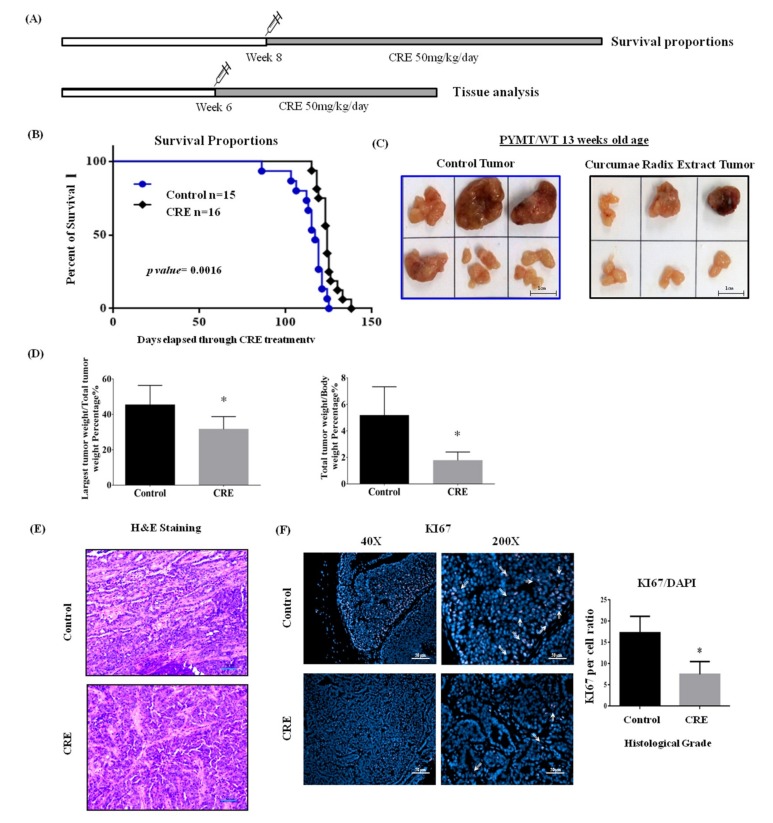
Ethanol extract of Curcumae radix prolongs survival rates of MMTV-PyMT transgenic mice. (**A**) For survival experiment, CRE administration was started at 8 weeks of age and continued until death of animal. For short term analysis, CRE administration was started at 6 weeks of age and mice were sacrificed at the beginning of 13 weeks. (**B**) Graphic represents Kaplan–Meier survival analysis over time with control vs. CRE treatment. The *p*-value was determined by using log–rank test. (**C**) Gross appearance of excised tumors at 13 weeks of age PyMT transgenic females with/without CRE treatment. Representative images of tumors excised from control and CRE group. Increased tumor size and larger necrotic areas were observed in the tumors excised from control group. (**D**) Total tumor weight and single largest tumor weight significantly decreased in CRE treated PyMT transgenic female mice. Values represent means ± S.E.M. * p < 0.05 vs. control. (**E**) Representative histological images taken of tumors derived from 13 weeks of age PyMT transgenic females with/without CRE treatment. Slides were microscopically examined and images taken. There was no significant difference at tumor grade and malignant progression between each group. Histologically, the tumors were carcinomas ranges from low to medium grade. Scale bars = 50 µm. (**F**) Curcumae radix extract suppressed cell proliferation in vivo. Immunohistochemistry staining against Ki67 in tumor tissues was done and representative images of tissue sections from each group were taken at 200× and 40× magnification. Scale bars = 50 µm. Histogram data was presented as mean ± SEM. * *p* < 0.05 vs. control.

Curcumae radix showed cytotoxicity and inhibited cell motility and migration in MCF7 cells. Its antitumor and anti-metastatic effects were further examined in vivo. To investigate the survival benefits, a dose of 50 mg/kg of CRE was administered orally five times in a week to PyMT female transgenic mice, starting from eight weeks of age until death (Figure 3A). As shown in Figure 3B, the extract of Curcumae radix prolonged the survival of MMTV-PyMT mice. Specifically, the median survival of CRE treated group was 68 days after the beginning of the treatment; whereas for the control group, the median survival was 61 days. The survival in CRE treated group was significantly longer than the one in the control group (*p* value = 0.0016). By day 69, all of the control mice were dead, whereas 25% of the CRE treated mice were still alive. The last mouse in the CRE treated group died at day 82. 

### 3.5. Curcumae Radix Extract Treatment Decreases Tumor Burden and Inhibits Cell Proliferation in Primary Tumor in PyMT Transgenic Mice

Female PyMTtransgenic mice from 6 to 13 weeks of age were treated with a dose of 50 mg/kg of CRE (Figure 3A). The excised tumors from the two groups appeared as light colored solid masses, consistent with adenocarcinoma. The tumor size of the primary tumor was significantly smaller in CRE treated group compared to control group (Figure 3C). Increased tumor size and larger necrotic areas were observed in the tumors excised from the control group. The total tumor weight and the single largest tumor weight were also significantly reduced in CRE treated group (Figure 3D). Tumor samples were examined histopathologically to determine whether CRE treatment affected the pathological progression of the primary tumors. Tumors excised from 13 week mice were observed and graded in accordance with the consensus report and recommendations put forth by the Annapolis pathology meeting about the mammary pathology of genetically engineered mice [33]. There was no significant difference at tumor grade and malignant progression between the two groups. Pathological grade of carcinomas were ranging from low to medium (Figure 3E). To further confirm that CRE decreased tumor burden, we examined Ki67 (cell cycle-specific antigen) for cell proliferation [34]. Immunohistochemical staining of Ki67 revealed that Curcumae radix extract treatment suppressed cell proliferation in primary tumors (Figure 3F).

### 3.6. Curcumae Radix Extract Treatment Interferes with Ccr7, Mmp9, c-Jun and c-Fos Expression Levels in Primary Tumor in PyMT Transgenic Mice

**Figure 4:**
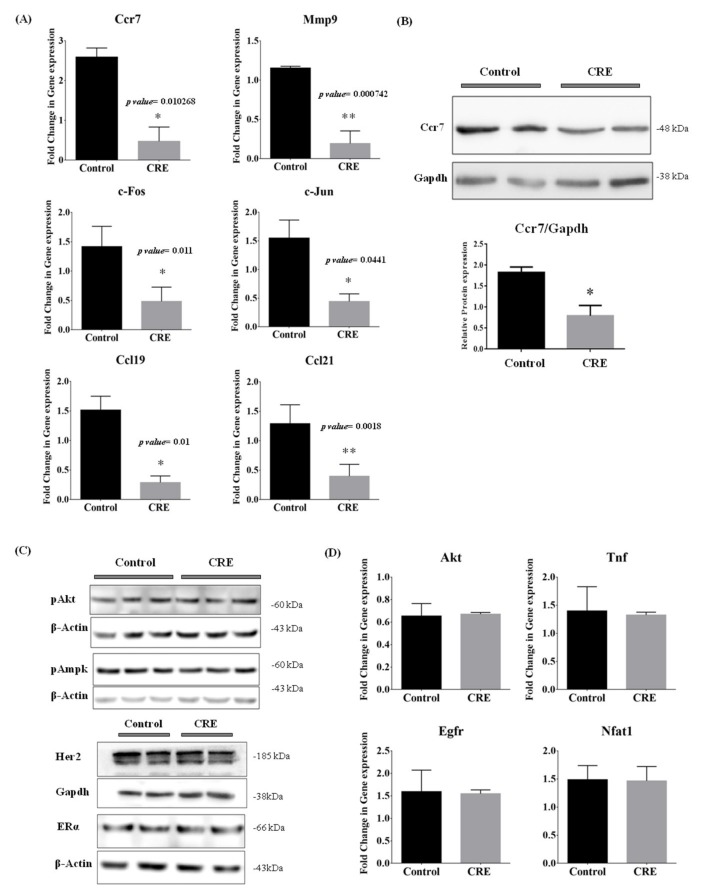
Curcumae radix extract treatment exhibited anti-metastatic effects on primary tumor of MMTV-PyMT transgenic mouse. (**A**) Quantitative analysis of real-time PCR was done using genes associated with breast cancer metastasis. The expression levels of *Ccr7, c-Fos, Mmp9, Ccl21,* and *Ccl19* were determined by qRT-PCR. The *Hprt* gene was used as input control, respectively, * *p* < 0.05 and ** *p* < 0.005 vs. control. (**B**) Western blot of proteins extracted from tumor tissues in the PyMT transgenic model against Ccr7 and Gapdh. Histogram data was presented as mean ± SEM. * *p* < 0.05 vs. control. (**C**) Western blot of proteins extracted from tumor tissues against Phosphorylated Akt, Phosphorylated Ampk, Her2, ERalpha, Gapdh and β-actin. (**D**) Quantitative analysis of real time PCR was done by using genes associated with apoptosis. The expression levels of *Jun, Akt*, and *Tnf* were determined by qRT-PCR. Data are representative of three independent experiments and are expressed as the mean ± SD.

We demonstrated that Curcumae radix extract inhibited cell migration by suppressing the expression of the metastasis-related genes *CCR7*, *MMP9*, *COX2* and their downstream genes, in MCF7 cells. Next, we further investigated whether these key regulators were affected by Curcumae radix extract in vivo. As shown in Figure 4A, the mRNA levels of Ccr7, Mmp9, c-Fos (Fos proto-oncogene, AP-1 transcription factor subunit), c-Jun (Jun), Ccl19 (C-C motif Chemokine Ligand 19) and Ccl21 were significantly reduced in tumor of CRE treated mice. c-Jun (Jun) and c-Fos assemble together to form the Activator Protein-1 (AP-1), which has a role in DNA binding, and acts as transcriptional activator during exponential growth [35]. Consistent with the mRNA result, also the protein level of Ccr7 was decreased upon CRE treatment (Figure 4B). Ccl19 has a receptor-ligand relationship with Ccr7, as well as Ccl21 [31]. The breast cancer markers Estrogen Receptor alpha (ERα) and Human Epidermal Growth Factor Receptor 2 (HER2) were examined at protein level (Figure 4C), however there was no significant difference between the two cohorts. Other key regulators for different signal pathways were also examined; however, no significant difference was found either at mRNA or protein levels (Figure 4C,D).

### 3.7. Curcumae Radix Extract Inhibits Mammary Tumor-Derived Lung Metastasis in PyMT Transgenic Mice

**Figure 5:**
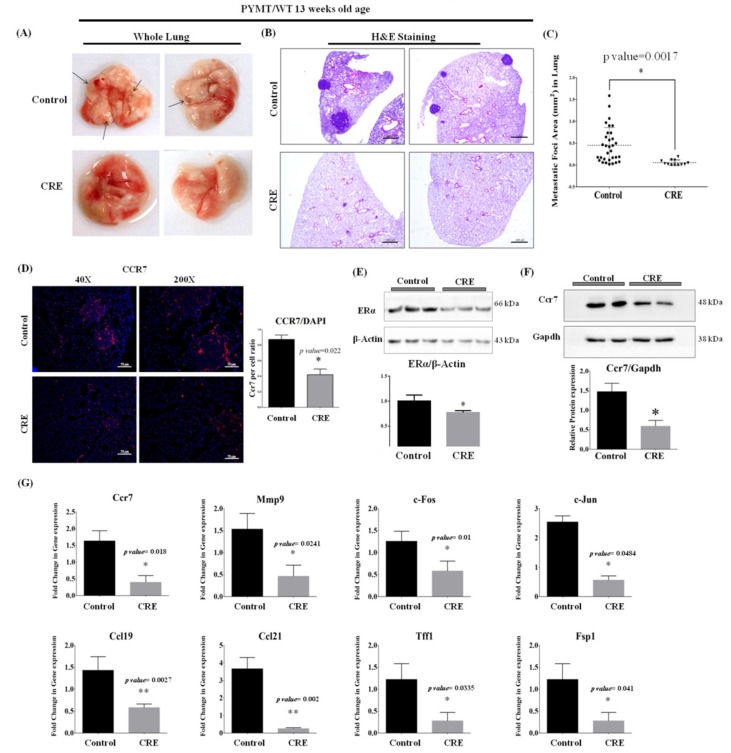
Gross and histological appearance of lung excised from 13 weeks of age PyMT transgenic females. CRE treated PyMT transgenic females showed decreased frequency of mammary tumor-derived lung metastases. (**A**) Representative photos of whole lung excised from 13 weeks age of control group (upper side) and CRE group (bottom side). Larger and more visible metastatic foci were observed in control group. (**B**) Hematoxylin and eosin (H&E) staining was done with lung samples excised from 13 weeks of age PyMT transgenic females with/without CRE treatment. Slides were microscopically examined and images taken. The number and size of metastases significant decreased in PyMT transgenic females with CRE treatment Scale bars = 400 µm. Curcumae radix extract suppresses frequency of PyMT mammary tumor-derived lung metastases and tumorigenesis by inhibiting CCR7 and its downstream genes in lung of PyMT transgenic mouse. (**C**) The distribution of sizes of metastatic foci in control group (*n* = 7) and CRE group (*n* = 6) lungs as mean ± SEM. * *p* < 0.05 vs. control. (**D**) Immunohistochemistry staining against Ccr7 in lung tissues was done and representative images of tissue sections from each group were taken at 200X and 40X magnification. Scale bars = 50µm. Histogram data was presented as mean ± SEM. * *p* < 0.05 vs. control. (**E**) Western blot of proteins extracted from lung tissues against ERalpha and β-actin. * *p* < 0.05 vs. control. (**F**) Level of Ccr7 protein in lung tissues was analyzed by Western blot. Gapdh was used as an internal control. * *p* < 0.05 vs. control. (**G**) To investigate level of transcripts associated with breast cancer metastasis the expression levels of Ccr7, c-Fos, Mmp9, Ccl21, Ccl19, Ps2, Fsp1 and Jun were monitored by qRT-PCR. β-actin gene was used as an internal control in real time PCR. Values represent means ± S.E.M. *n* = (3); * *p* < 0.05 and ** *p* < 0.005 vs. control.

Our MMTV-PyMT mouse model provides lots of advantages to examine mammary tumor-derived lung metastasis. All of the used PyMT transgenic mice developed lung metastases and this could be observed even by naked eye in 16 week-old mice. Here, we decided to examine PyMT mice at 13 weeks of age to evaluate the anti-metastatic effect of Curcumae radix extract. Overall, the structure and the colors of the excised lung lobes appeared similar between the two groups. However, as shown in Figure 5A, small bead-like lung metastases were observed in excised lungs of control group (upper image), while no bead-like structures were observed in excised lungs of CRE group (bottom image). Microscopically, there were significant differences between lung metastases derived from the control and the CRE treated group. Specifically, the size of the lung metastases generally appeared greater in control group lobes (Figure 5B upper image) than in CRE treated group (Figure 5B bottom image). As shown in Figure 5C, there was a significant difference in the area of metastatic foci between the control and CRE group lungs (*p*-value < 0.001). The size of the metastatic foci in the lungs excised from control group ranged from 0.02 to 1.588 mm^2^, with a mean value of 0.45 ± 0.07 mm^2^, whereas those excised from CRE treated group ranged from 0.055 ± 0.017 mm^2^, with a mean value of −0.397 ± 0.1187 mm^2^. Lower Ccr7 levels were observed in lung excised from CRE treated mice (Figure 5D). Since, the size of the lung metastases generally appeared greater in control group lobes, smaller tumors from control group were selected to analyze for better comparison.

Further analyses were conducted in lung samples to investigate the signal pathways which might be affected by Curcumae radix extract. During the early stage of breast cancer, the over-expression of estrogen receptor alpha (ERα) is frequently observed [36]. In addition, it is known that the estrogen receptor over-expression induces tumor cell proliferation [37]. Thus, it can be concluded that ERα levels and tumor ratio are directly proportional. As shown in Figure 5E, the protein level of ERα was dramatically decreased in lung excised from CRE treated group indicates that tumor ratio is lower than the control group. Curcumae radix extract suppressed the metastasis from primary tumor cells, causing smaller and less frequent lung metastasis and naturally decreased ERα level. Curcumae radix extract treatment also decreased the protein level (Figure 5F) and the mRNA level (Figure 5G) of the Ccr7. 

As shown in Figure 5G, the expression levels of Mmp9, c-Fos, c-Jun, Ccl19, Ccl21, Fsp1 (S100 calcium binding protein A4, S100A4) and Tff1 (trefoil factor 1) were significantly reduced in lung of CRE treated mice. FSP1 (S100A4) promotes breast cancer metastasis by the induction of EMT (epithelial-mesenchymal transition) [38]. It has been reported that TFF1 mRNA level is higher in blood and in ER positive primary tumor in breast cancer patients with metastatic disease compared with the patients without metastasis. It can be said that TFF1 can be used as a marker in breast cancer tumorigenesis, as the detection of TFF1 mRNA in cells isolated from the peripheral blood can contribute to the prediction of endocrine response [39]. These results indicated that Curcumae radix (turmeric root) extract inhibits the gene expression of metastasis related genes.

### 3.8. CCR7 Regulates the Gene Expression of CCL21, MMP9 and AP1 Complex (c-FOS and c-JUN)

**Figure 6:**
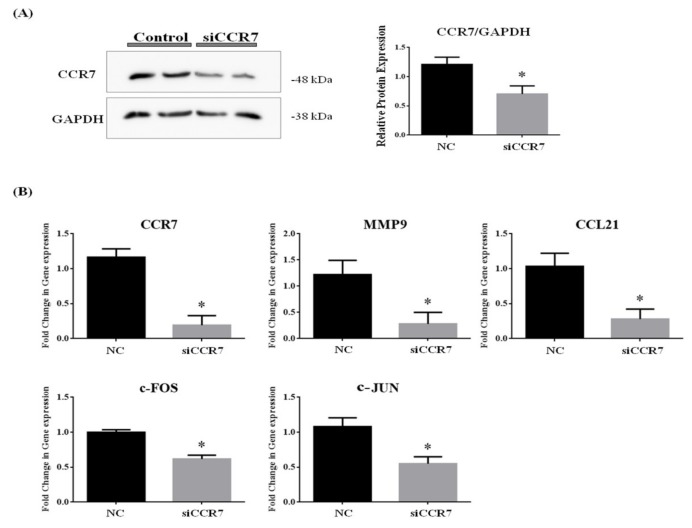
Downregulation of *CCR7* gene by siRNA inhibits gene expression of MMP9, c-FOS, c-JUN and CCL21 in vitro. (**A**) Western blot of proteins extracted from MCF7 cell against CCR7 and GAPDH. (**B**)To investigate level of transcripts associated with CCR7, MMP9, CCR7, c-FOS, c-JUN and CCL21 were monitored in MCF7 cell transfected with CCR7 siRNA by using qPCR. Β-ACTIN and 18S mRNA was used as an internal control in real time PCR. Data are representative of four independent experiments and are expressed as the mean ± SD; * *p* < 0.05 vs. negative control.

We investigated the correlation between CCR7, CCL21, MMP9 and AP1 complex (c-FOS and c-JUN) gene expressions. The effect of CCR7 siRNA treatment on protein expression was assessed by Western blot analysis. As shown in Figure 6A, the band density clearly decreased in CCR7 siRNA-treated group compared with the control group. We demonstrated that siRNA targeting CCR7 significantly downregulated CCR7 protein expression in MCF7 cell. Small interfering RNA mediated down regulation of CCR7 gene expression led to a dramatic decrease in mRNA levels of MMP9, c-FOS (AP1), c-JUN (AP1) and CCL21 (Figure 6B). These results indicated that suppressing CCR7 leads to downregulation of MMP9 and AP1 complex (c-FOS and c-JUN) gene expression.

## 4. Discussion

Metastatic breast cancer represents the major clinical problem of cancer treatment that cannot be removed easily by surgery compared with the primary tumor, due to its unusual and distinct pattern. The incidence of breast cancer and its mortality rate are steadily increasing. Although several target molecules have been reported, the precise mechanism is still not known due to complex interactions between primary tumor cells and tumor-associated stroma. Furthermore, currently available medications cause numerous side effects without improving the quality of life and patients’ survival. Therefore, non-toxic and effective treatment agents are needed for treating metastatic breast cancer. 

Curcumae radix is the dry root of *Curcuma longa L.* (turmeric) that contains numerous chemical entities and multiple active components including curcumin, demethoxycurcumin, and bis-demethoxycurcumin. It has been demonstrated in several number of studies that curcumin has anticancer, anti-proliferative, and anti-inflammatory properties [40]. However, besides curcumin, Turmeric contains numerous other chemical components that exert anti-inflammatory and anticancer activities, such as turmerone, elemene and furanodiene [41] and the combination of these compounds enhances their bioavailability [42]. In the present study, Curcumae radix ethanol extract was chosen as the subject of the study, instead of using a turmeric compound alone. In addition, the effect of curcumin on breast cancer has been demonstrated in several studies, but the effect of Curcumae radix (dried turmeric root) extract on breast cancer is still unknown. 

In this study, we have shown that Curcumae radix extract significantly enhances survival of MMTV-PyMT female transgenic mouse model. We examined the tumors focusing on the development of breast cancer, as well as on lung metastasis, by utilizing the PyMT transgenic mice. A striking finding is that CRE increased the median survival of MMTV-PyMT mice by 7 days and prolonged survival without any side effects. Moreover, tumor burden was substantially decreased in CRE treated PyMT transgenic mice, with up to ∼60% decrease in combined tumor weight per mouse. The PyMT transgenic mice control group showed significant increases in tumor burden, multiplicity and necrotic areas, compared with the ones in the CRE-treated group. Larger necrotic areas are likely to be due to the increased size of the tumors, as the tumor could not keep pace with the rapidly proliferating tumor cells, which lost the ability of tumor angiogenesis and oxygen supply [43]. This finding can be supported by the decreased levels of cell proliferation marker (Ki67) protein in CRE treated tumors, which means that there is less proliferation and naturally smaller necrotic areas. Morphologically, tumors derived from PyMT mice, either with or without CRE treatment; do not show any differences, indicating that CRE does not affect the histopathological progression of primary tumor at 13 weeks of age. 

The suppression of mammary tumor-derived lung metastasis is another striking effect of Curcumae radix extract in breast cancer. Significant differences were observed histopathologically and morphologically, between lung metastases derived from control and CRE treated groups. The size of the lung metastases generally appeared larger in control group lobes than in CRE treated group. The dramatic decrease in Estrogen Receptor alpha protein levels may indicate that CRE suppressed metastasis of primary tumor and may be linked with the smaller size of lung metastases. 

Interestingly, CRE treatment decreased the mRNA expression levels of metastasis markers in both lung and primary tumor samples. In vitro results were consistent with in vivo analysis. We investigated several genes that mark and mediate breast cancer metastasis providing growth advantages, both in primary tumor and tumor-associated stroma. We noticed that CCR7, MMP9, c-FOS, c-JUN (JUN) and CCL21 mRNA expressions were affected by Curcumae radix extract treatment, both in vivo and in vitro. The key regulators for different signal pathways were also evaluated; however, there was no significant change at mRNA levels.

The Chemokine Receptor *CCR7* is necessary for the regulation of chemotaxis and migratory speed [44]. The over-expression of CCR7 promotes the capacity of EMT cells to migrate toward CCL21-expressing tissue and to disseminate through the lymphatic system [45]. It has been reported that COX2 and NF-κB are important for the regulation of CCR7 expression and that NF-κB binding sites were found in the *CCR7* gene [45]. However, Curcumae radix did not change NF-κB mRNA levels in our study. In addition, although CRE treatment decreased COX2 mRNA level in vitro, we could not observe the same results in vivo. Thus, we focused on the correlation between CCR7 and AP1 complex in breast cancer. It has been reported that *CCR7* gene play an important role in cancer metastasis [46] and even *CCR7* has been shown as a novel biomarker that can predict lymph node metastases in breast cancer [47]. In a recent study, researchers examined role of *CCR7* gene as a metastasis and prognosis indicator in patients with esophageal carcinoma. They found that CCR7 expression was significantly associated with T stage and lymph node metastasis. It has been mentioned that the 5-year survival rate was significantly lower in patients with CCR7 expression compared to those without [48]. In our study, we also demonstrated that CRE significantly prolongs allover survival of PyMT transgenic mouse. In another study, CCR7 knockdown by siRNA significantly abrogated the CCL21-induced migration of transitional cell carcinoma UM-UC-3 cells. The group designed a wound healing assay that first induced migration of cells by CCL21 supplement. While cell migration was induced in control cells, siCCR7-transfected cells suppressed metastasis even in the presence of an inducer [49]. In our study, we also demonstrated that CRE significantly suppressed cell motility and cell migration by classic wound healing assay. Thus, we concluded that *CCR7*gene could be a possible target of CRE for decreasing lung metastasis of the primary tumor.

Activator Protein-1 (AP1) plays a role in tumorigenesis; specifically, the over-expression of Jun (c-Jun) and c-Fos is able to transform normal cells into cancer cells [4,35]. It has been shown that AP1 complex plays a role in the regulation of CCR7 expression in metastatic squamous cell carcinoma cells [50] and gallbladder cancer cells [51]. On the other side, some studies indicated that CCR7/CCL21 interaction regulates MMP9 gene transcription by regulating Extracellular signal-related kinase (ERK)-effector c-Fos (AP1 complex) in B-cell chronic lymphocytic leukemia cell [52]. Moreover, another study indicates that the disruption of the *CCR7* gene impairs AP1 transcriptional activity by inhibiting the extracellular signal-related kinase (ERK) and c-JUN phosphorylation in Forkhead box P3 (Foxp3^+^) regulatory T-Cells [53]. The interaction between CCR7 and AP1 complex in breast cancer is not clear. Here, we demonstrated that AP1 and MMP9 expressions in breast cancer cells were positively correlated with the levels of CCR7 and the downregulation of CCR7 reduced the mRNA levels of MMP9 and AP1 complex (c-Fos and c-Jun). 

In conclusion, we revealed that Curcumae radix suppresses the level of CCR7 and inhibits migration and metastasis of breast cancer cells to lung, as well as downregulates AP1 and MMP9 expression levels. The results of our study suggest that CCR7 mediates the migration of tumor cells may be through the “CCR7–AP1–MMP9” pathway. The present study demonstrated for the first time that Curcumae radix (turmeric root) extract treatment could suppress breast cancer tumor growth and inhibit lung metastasis in a MMTV-PyMT transgenic mouse model. Curcumae radix exhibited antitumor and anti-metastatic effects and these results highlight the potential of Curcumae radix as a resource for treatment and prevention of breast cancer metastasis.

## Figures and Tables

**Table 1:** Primers used for real-time or conventional PCR.

Gene	Forward Primer	Reverse Primer	Species
***CCR7***	*TGA GGT CAC GGA CGA TTA CAT*	*GTA GGC CCA CGA AAC AAA TGA T*	*Human*
***c-JUN***	*TCC AAG TGC CGA AAA AGG AAG*	*CGA GTT CTG AGC TTT CAA GGT*	*Human*
***c-FOS***	*CCG GGG ATA GCC TCT CTT ACT*	*CCA GGT CCG TGC AGA AGT C*	*Human*
***HER2***	*TGT GAC TGC CTG TCC CTA CAA*	*CCA GAC CAT AGC ACA CTC GG*	*Human*
***MMP9***	*TGT ACC GCT ATG GTT ACA CTC G*	*GGC AGG GAC AGT TGC TTC T*	*Human*
***CCL19***	*CTG CTG GTT CTC TGG ACT TCC*	*AGG GAT GGG TTT CTG GGT CA*	*Human*
***CCL21***	*GTT GCC TCAA GTA CAG CCA AA*	*AGA ACA GGA TAG CTG GGA TGG*	*Human*
***NFAT1***	*GAG CCG AAT GCA CAT AAG GTC*	*CCA GAG AGA CTA GCA AGG GG*	*Human*
***COX-2***	*CTG GCG CTC AGC CAT ACA G*	*CGC ACT TAT ACT GGT CAA ATC CC*	*Human*
***TNF***	*CCT CTC TCT AAT CAG CCC TCT G*	*GAG GAC CTG GGA GTA GAT GAG*	*Human*
***Ccr7***	*TGT ACG AGT CGG TGT GCT TC*	*GGT AGG TAT CCG TCA TGG TCT TG*	*Mouse*
***c-Fo*** ***s***	*CGG GTT TCA ACG CCG ACT A*	*CGG GTT TCA ACG CCG ACT A*	*Mouse*
***c-*** ***Jun***	*CCT TCT ACG ACG ATG CCC TC*	*GGT TCA AGG TCA TGC TCT GTT T*	*Mouse*
***Her2***	*ACC GAC ATG AAG TTG CGA CTC*	*AGG TAA GCT CCA AAT TGC CCT*	*Mouse*
***Mmp9***	*CTG GAC AGC CAG ACA CTA AAG*	*CTC GCG GCA AGT CTT CAG AG*	*Mouse*
***Ccl19***	*GGG GTG CTA ATG ATG CGG AA*	*CCT TAG TGT GGT GAA CAC AAC A*	*Mouse*
***Ccl21***	*GTG ATG GAG GGG GTC AGG A*	*GGG ATG GGA CAG CCT AAA CT*	*Mouse*
***Fsp1***	*TCC ACA AAT ACT CAG GCA AAG AG*	*GCA GCT CCC TGG TCA GTA G*	*Mouse*
***Ps2***	*AGC ACA AGG TGA TCT GTG TCC*	*GGA AGC CAC AAT TTA TCC TCT CC*	*Mouse*
***Tnf***	*CCT GTA GCC CAC GTC GTA G*	*GGG AGT AGA CAA GGT ACA ACC C*	*Mouse*
***Akt***	*ATG AAC GAC GTA GCC ATT GTG*	*TTG TAG CCA ATA AAG GTG CCA T*	*Mouse*
***Cox-2***	*TTC AAC ACA CTC TAT CAC TGG C*	*AGA AGC GTT TGC GGT ACT CAT*	*Mouse*
***β-ACTIN***	*CAT GTA CGT TGC TAT CCA GGC*	*CTC CTT AAT GTC ACG CAC GAT*	*Human*
***GAPDH***	*GGA GCG AGA TCC CTC CAA AAT*	*GGC TGT TGT CAT ACT TCT CAT GG*	*Human*
***Hprt***	*AGG CCC CAA AAT GGT TAA GGT T*	*CAA GGG CAT ATC CAA CAA CAA A*	*Mouse*
***β-Actin***	*GGC TGT ATT CCC CTC CAT CG*	*CCA GTT GGT AAC AAT GCC ATG T*	*Mouse*

**Table 2:** The marker compound, linear ranges, regression equation, and correlation coefficient of the marker compounds in Curcumae Radix.

Compound	Retention Time (min)	Linear Range (μg/mL)	Regression Equation	Correlation Coefficient (*r*^2^)	Amount (μg/mg) *
curcumin	17.60	3.125–400	y = 20.2393x − 30.2014	0.999	15.28 ± 0.097
demethoxycurcumin	16.95	3.125–400	y = 22.7311x − 39.6534	0.999	3.82 ± 0.037
bisdemethoxycurcumin	16.32	3.125–400	y = 59.2165x − 27.5719	0.999	0.63 ± 0.010

* Mean ± SD (SD is the standard deviation); y: peak area (mAU) of compounds; x: concentration (μg/mL) of compounds.
